# The White Matter Hyperintensity Shape and Brain Clearance (WHIMAS) Study for Identification of Novel 7T Magnetic Resonance Imaging Markers of Cerebral Small Vessel Disease: Protocol for a Cross-Sectional Study

**DOI:** 10.2196/77681

**Published:** 2025-12-04

**Authors:** Jasmin Annica Kuhn-Keller, Ingmar Eiling, Lydiane Hirschler, Lena Václavů, Marie-Noëlle Witjes-Ané, Marjolein Wijngaarden, Martijn Nagtegaal, Ece Ercan, Nathaly Rius Ottenheim, Marjan van der Elst, Evelien Sohl, Mark A van Buchem, Simon Mooijaart, Matthias JP van Osch, Jeroen de Bresser

**Affiliations:** 1 CJ Gorter MRI Center Department of Radiology Leiden University Medical Center Leiden, South Holland The Netherlands; 2 Department of Radiology Leiden University Medical Center Leiden, South Holland The Netherlands; 3 Department of Internal Medicine Section of Gerontology and Geriatrics Leiden University Medical Center Leiden, South Holland The Netherlands; 4 Department of Psychiatry Leiden University Medical Center Leiden, South Holland The Netherlands; 5 Center for Medicine for Older People Leiden University Medical Center Leiden, South Holland The Netherlands; 6 Department of Geriatrics Alrijne Hospital Leiden Leiden, South Holland The Netherlands

**Keywords:** cerebral small vessel disease, dementia, cognitive impairment, brain clearance, magnetic resonance imaging

## Abstract

**Background:**

Sporadic cerebral small vessel disease (SVD) has a heterogeneous underlying pathology, and current SVD magnetic resonance imaging (MRI) markers do not accurately capture this heterogeneity. Novel ultrahigh-field (7T) brain MRI markers provide a window of opportunity to study early changes and potential determinants of SVD. White matter hyperintensity (WMH) shape is a relatively novel MRI marker of SVD and has shown prognostic potential. However, the exact microstructural changes within or surrounding WMHs or potential causes related to WMH shape variations are unknown. Furthermore, impaired brain clearance via the recently discovered brain clearance system may be another early change or potential cause of SVD.

**Objective:**

In the White Matter Hyperintensity Shape and Brain Clearance (WHIMAS) study, we aim to assess the link between WMHs—their shape in particular—and brain clearance and other MRI markers on ultrahigh-field (7T) brain MRI and show whether these markers are associated with cognitive functioning in older adults with memory complaints.

**Methods:**

This is a cross-sectional study conducted at the Leiden University Medical Center. A total of 50 outpatients from the memory or geriatric clinic aged ≥65 years will be recruited for a 3T and 7T MRI scan (including clinical structural scans, eg, 3D T1-weighted, 3D fluid-attenuated inversion recovery), and experimental scans such as cerebrospinal fluid (CSF)–selective T2-prepared readout with acceleration and mobility encoding (CSF-STREAM) and the relationship between blood oxygen level–dependent [BOLD] signals and CSF flow) and magnetic resonance fingerprinting, as well as a standardized neuropsychological test battery (domains: memory, executive function, visuoconstruction, and processing speed). We will assess WMH shape markers (solidity, convexity, concavity index, fractal dimension, and eccentricity) and brain clearance markers (CSF mobility and the relationship between blood oxygen level–dependent signals and CSF flow) and study their relationship to other MRI markers and cognitive functioning using multivariable regression analyses.

**Results:**

Patient inclusion started in January 2023, and study enrollment of patients is expected to finish in the second quarter of 2027, whereas the main results are expected to be published in the first quarter of 2028.

**Conclusions:**

We aim to understand variations in WMH shape and find their relationship to cerebral SVD and markers of brain clearance and cognitive functioning. WMH shape and brain clearance markers early in the disease process of SVD are extremely important as they may represent a basis for future patient selection for lifestyle interventions or for treatment trials aimed at the prevention of dementia.

**Trial Registration:**

ClinicalTrials.gov NCT06010511; https://clinicaltrials.gov/study/NCT06010511

**International Registered Report Identifier (IRRID):**

DERR1-10.2196/77681

## Introduction

Dementia is often characterized by a combination of neurovascular and neurodegenerative disease processes, and mixed pathologies are common [1]. The most common mixed pathology is Alzheimer dementia and cerebral small vessel disease (SVD) [2,3]. SVD contributes to the clinical phenotype of dementia in approximately 45% of cases [2-4]. There are 2 main types of SVD in an aging population. One is SVD due to arteriosclerosis, often related to hypertension [5]. The other one is cerebral amyloid angiopathy, characterized by progressive amyloid accumulation in the vessel walls [5]. Current SVD markers such as white matter hyperintensities (WMHs) of presumed vascular origin are unspecific, and they fail to accurately capture the heterogeneity of SVD pathology. Therefore, novel brain magnetic resonance imaging (MRI) markers are needed to identify early changes and potential determinants of SVD. These markers are extremely important early in the disease process of SVD as they may represent a basis for future patient selection for lifestyle interventions or outcome markers for treatment trials such as those currently being developed for cerebral amyloid angiopathy [6] aimed at the prevention of dementia.

WMHs are the key brain MRI manifestation of cerebral SVD [7]. Approximately 92% of all individuals aged >60 years have WMHs [8-10], and a higher WMH burden is a risk factor for occurrence of stroke and dementia [10]. In particular, the volume of WMHs has been extensively studied, but this is a generic and nonspecific marker that only has modest prognostic value [11]. Although there is considerable variation in the shape of WMHs, this marker has received little attention [12]. Recent studies have shown that normal-appearing white matter around WMHs that progressed on follow-up MRI scans already showed changes in structural integrity and hemodynamics at baseline [13,14]. Furthermore, WMHs typically localize at vascular endzones and progress along more proximal parts of the perforating arteries [15]. How the perforating arteries are affected depends on the underlying pathological changes, such as large vessel atheromas at the origin of perforating arteries, small vessel atheromas, or microembolisms [16,17]. These pathological changes may lead to hypoperfusion, defective cerebrovascular reactivity, and blood-brain barrier dysfunction [11], which in turn may be related to increase in WMHs and, thus, also to changes in their shape [12,17]. Previous studies have also indicated that WMH shape may provide a better indication of underlying pathophysiological mechanisms than WMH volume alone and may harbor strong prognostically relevant information [12,18-20].

SVD is a heterogeneous disease with many possible underlying causes. An important factor leading to brain changes in SVD might be impaired clearance of waste products, which has been linked to aging and dementia pathology [7]. The process of brain clearance is postulated to be partly driven by the brain clearance system, where cerebrospinal fluid (CSF) and interstitial fluid “flush” brain tissue and transport waste products out of the brain via perivascular spaces (PVS). Some first studies have shown that, in cerebral amyloid angiopathy and Alzheimer dementia, brain clearance function might be impaired [21]. Currently, brain clearance–related processes can only be studied invasively in humans, for example, through contrast-enhanced MRI following intrathecal injection [22]. This limitation makes it difficult to implement research related to brain clearance dysfunction into clinical study protocols. However, recently developed novel ultrahigh-field (7T) brain MRI markers provide a window of opportunity to study the human brain clearance system in a noninvasive way.

In the White Matter Hyperintensity Shape and Brain Clearance (WHIMAS) study, we aim to study the link between WMHs, and especially their shape, and brain clearance and other MRI markers on high-field (3T) and ultrahigh-field (7T) brain MRI. Furthermore, we aim to study the relationship between WMH shape and brain clearance markers (eg, the relationship between blood oxygen level–dependent [BOLD] signals and CSF flow, also referred to as BOLD-CSF coupling, and CSF mobility) and cognitive functioning (domains: memory, executive function, visuoconstruction, and processing speed).

Studying WMH shape and brain clearance markers in SVD is important because, at an early stage, cerebral SVD is a target for preventive treatment that may postpone or even prevent the occurrence of dementia and stroke. Our study contributes to research into early detection of dementia by first investigating potential markers in a population with the disease to identify markers that might also be relevant in larger studies in healthy older individuals. We specifically strive for early detection of the potentially treatable or modifiable part underlying the dementia phenotype, namely, SVD. Previous studies have already shown that early lifestyle interventions in populations at risk can slow the pathophysiological processes of cerebral SVD [23-25]. However, it is currently impossible to conduct early identification of individuals who have an increased risk of dementia, who may benefit most from available lifestyle interventions or from inclusion in treatment trials aimed at prevention. With our study, we aim to pave the way for personalized medicine approaches by finding brain MRI biomarkers that, in the future, can identify these individuals at increased risk of dementia based on SVD burden.

## Methods

### Study Design

**Figure 1:**
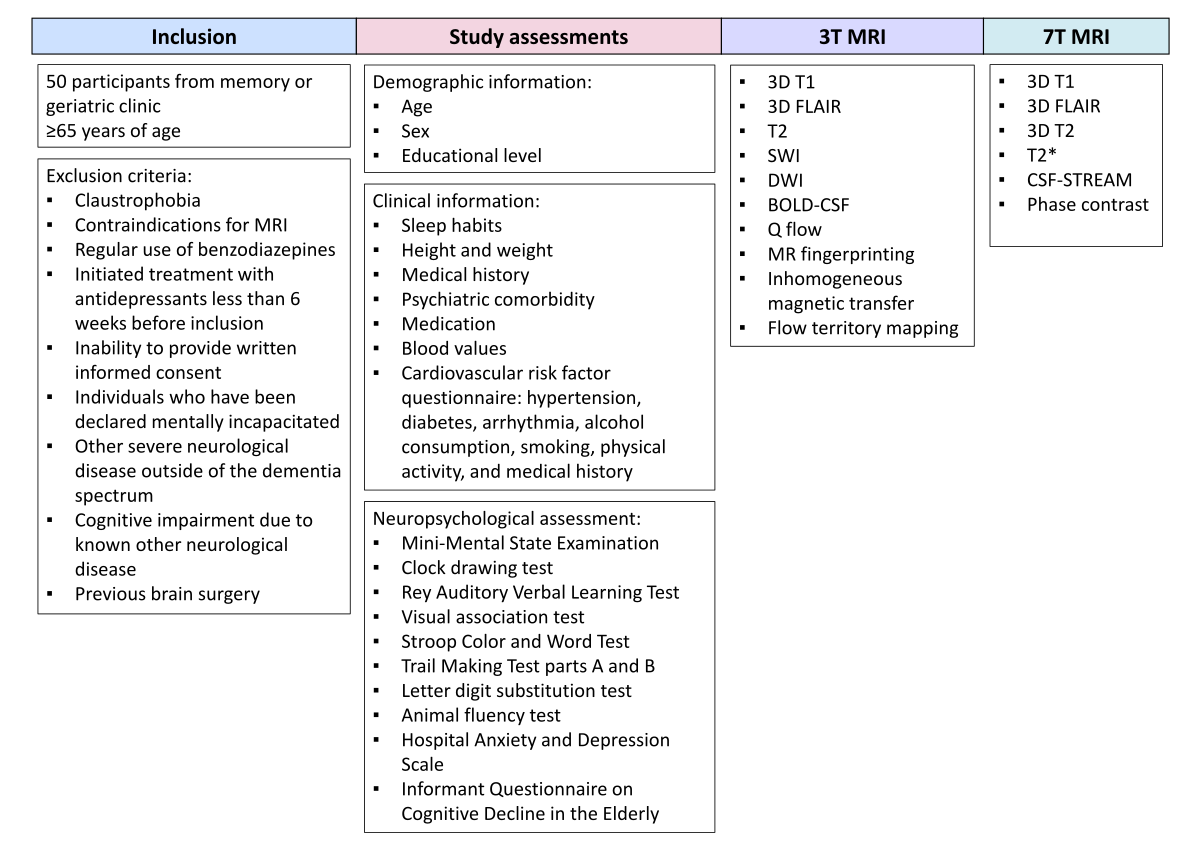
Overview of the study procedures. 3D T1: T1-weighted scan; BOLD-CSF: blood oxygen level–dependent signal and cerebrospinal fluid coupling; CSF-STREAM: cerebrospinal fluid–selective T2-prepared readout with acceleration and mobility encoding; DWI: diffusion-weighted scan; FLAIR: fluid-attenuated inversion recovery; MR: magnetic resonance; MRI: magnetic resonance imaging; SWI: susceptibility-weighted scan; T2: T2-weighted scan.

This is an observational cross-sectional study that will be conducted at the Leiden University Medical Center (LUMC). This study involves a whole-day visit to the LUMC for each participant and includes the following procedures: a 3T brain MRI scan of 60 minutes, a 7T brain MRI scan of 60 minutes, a neuropsychological assessment, and questionnaires on demographics and vascular risk factors. An overview of the study procedures can be found in Figure 1. The objectives of this study are as follows: (1) to study the association between a more irregular WMH shape and anatomical, hemodynamic, and white matter integrity abnormalities on MRI; (2) to study the association between WMH shape and cognition or other cerebral SVD markers; and (3) to study the association between novel MRI markers of brain clearance and both cerebral SVD markers and cognition.

### Population

We will prospectively include 50 outpatients aged >65 years with memory complaints from the memory or geriatric clinic in one of their first visits to LUMC (Leiden, the Netherlands), Alrijne Hospital (Leiden, the Netherlands), or Haga Hospital (The Hague, the Netherlands). All participants provide written informed consent before any study procedures. This study involves 3T and 7T MRI and newly developed sequences and markers, especially for brain clearance, which have not been applied in patient populations before. Therefore, we conducted the sample size calculation based on the WMH shape analysis. To provide a frame of reference, we performed a sample size calculation using data from a previous study [26]. The linear regression on hypertension and convexity, corrected for age and sex, was conducted using data from 71 older adults without dementia. The same WMH shape analysis pipeline was used in the previous study as we aim to use it in this study. Using the data from the significant results of the aforementioned study, the sample size calculation conducted in G*Power [27] (input parameters: effect size=0.1; α=.05) showed a result of 79 participants. As the data used in the calculation were obtained from community-dwelling individuals, we expect higher effect sizes in our study, which comprises a memory clinic population. In this population, the prevalence and severity of SVD and, thus, WMHs will be higher, justifying our assumption of increased power. At the same time, we do not want to risk an underpowered study. For example, in a case-control study comparing patients with type 2 diabetes with healthy controls, an average effect size of 0.37 for a more irregular WMH shape was found [12]. To illustrate this, we calculated the sample size with 2 different effect sizes that are higher than the one used in the initial calculation (0.2 and 0.3; α remained at .05) but still lower than 0.37 (as found by de Bresser et al [12]). These calculations resulted in a sample size of 42 or 29 participants, which was obtained using the effect sizes of 0.2 and 0.3, respectively. Therefore, 50 participants should provide the necessary statistical power to overcome biological and clinical variation.

To be eligible to take part in this study, a participant must meet all the following criteria: receipt of care in the outpatient memory clinic or the geriatric clinic at LUMC or the memory clinic at Alrijne Hospital or Haga Hospital, age of >65 years, and eligibility for MRI. Moreover, the participants must have native-level proficiency in Dutch due to the requirements of the neuropsychological assessment.

A potential participant who meets any of the following criteria will be excluded from this study: (1) claustrophobia; (2) contraindications for MRI, such as metal implants or pacemakers; (3) regular use of benzodiazepines; (4) initiation of treatment with antidepressants less than 6 weeks before inclusion; (5) inability to provide written informed consent (assessed by the treating physician); (6) individuals who have been declared mentally incapacitated; (7) other severe neurological disease outside of the dementia spectrum; (8) cognitive impairment due to known other neurological diseases; and (9) previous brain surgery.

### Clinical Data

We will collect basic demographic information, including age, sex, and educational level. Information about medical history, psychiatric comorbidity, medication, and current blood values are extracted from the clinical files of the participants. Furthermore, a cardiovascular risk factor questionnaire will be used to gather information about hypertension, diabetes, arrythmia, alcohol consumption, smoking, physical activity, and medical history. We will also assess sleep habits using an adapted version of the Pittsburg Sleep Quality Index [28]. The sleep questionnaire will be included because of the influence of sleep on the brain clearance system.

The following tests will be included in the neuropsychiatric assessment: (1) Mini-Mental State Examination [29], (2) clock drawing test [30], (3) 15-Word Verbal Learning Test (immediate and delayed) [31,32], (4) visual association test [33], (5) Stroop Color and Word Test (40-item version) [34], (6) Trail Making Test parts A and B [35,36], (7) letter digit substitution test [37], (8) animal fluency test [38], (9) Hospital Anxiety and Depression Scale [39], and (10) Informant Questionnaire on Cognitive Decline in the Elderly [40].

### MRI Scans

All MRI scans will be conducted at the LUMC using a 3T Philips Ingenia Elition and a 7T Philips Achieva MRI scanner (Philips Healthcare). The MRI scan protocols are shown in Table 1.

**Table 1:** Magnetic resonance imaging (MRI) scan protocols on the 3T and 7T MRI scanners.

			Purpose		Parameters		Acquisition resolution (mm^3^)		FOV^a^ (mm^3^)		Acquisition time		Additional information
**3T MRI**													
	3D MPRAGE^b^ (T1 weighted)^c^	Anatomical information		TR^d^=9.9 ms and TE^e^=4.6 ms		1 mm isotropic		256 × 256 × 170		4 min, 20 s		FA^f^=8°	
	3D FLAIR^c,g^	WMH^h^ and lacunes		TR=4800 ms and TI^i^=1650 ms		1 mm isotropic		220 × 220 × 175		4 min, 48 s		Refocusing angle=40°	
	2D TSE^j^ (T2 weighted)^c^	Perivascular spaces and arteries		TR=4490 ms and TE=80 ms		0.40 × 0.50 × 3.00		230 × 183 × 150		2 min, 15 s		FA=90°	
	3D SWI^c,k^	Iron, microbleeds, and superficial siderosis		TR=31 ms and TE=7.20 ms		0.60 × 0.60 × 2.00		220 × 182 × 130		2 min, 35 s		FA=17°	
	2D DWI^c,l^	Acute or recent ischemic lesions		TR=3206 ms and TE=67 ms		1.96 × 2.44 × 5.00		220 × 220 × 148		38 s		b-value=0, 1000	
	BOLD-CSF^c,m^	CSF^n^ hemodynamics coupling		TR=370 ms and TE=25 ms		2.88 × 2.88 × 5.50		230 × 230 × 115		5 min, 7 s		FA=40°	
	2D phase contrast (Q flow)^c^	Large vessel flow		TR=15 ms and TE=7.2 ms		0.45 × 0.45 × 4.00		230 × 230		1 min, 2 s		Velocity encoding value=80 cm/s; planned using very fast phase-contrast angiograms	
	MR^o^ fingerprinting	White matter structural integrity; myelin water imaging		TR=15 ms and TE=3.0 ms		1.00 × 1.00 × 4.00		240 × 240		5 min		FA range=10.0-60.0°; requires B1+ map	
	Inhomogeneous magnetization transfer (3D)	White matter structural integrity		TR=90 ms and TE=1.31 ms		2.29 × 2.29 × 5.00		220 × 220 × 96		7 min, 17 s		FA=7°; custom off-resonance parameters	
	Flow territory mapping	Perfusion territory scan		TR=4550 ms and TE=16 ms		2.50 × 2.56 × 5.00		240 × 240 × 96		4 min, 56 s		pCASL^p^	
**7T MRI**													
	MPRAGE (T1 weighted)^c^	Anatomical information		TR=4.2 ms and TE=1.9 ms		0.90 mm isotropic		246 × 246 × 174		2 min, 22 s		FA=7°	
	3D FLAIR^c^	WMHs and lacunes		TR=8000 ms and TI=2200 ms; TE=252 ms		0.70 × 0.70 × 1.40		240 × 209 × 180		5 min, 12 s		FA=90°	
	3D TSE (T2 weighted)^c^	Perivascular spaces and arteries		TR=3000 ms and TE=300 ms		0.75 mm isotropic		250 × 250 × 190		4 min, 6 s		FA=100°	
	T2*-weighted GRE^q^	Iron, microbleeds, superficial siderosis, and veins		TR=1830 ms and TE=25 ms		0.24 × 0.24 × 1.00		240 × 180 × 101		10 min, 32 s		FA=60°	
	CSF-STREAM^r^	Whole-brain CSF dynamics		TR=3430 ms and TE=497 ms		0.45 mm isotropic		250 × 250 × 190		21 min, 57 s		FA=90°	
	2D real-time phase contrast (2x)	Blood and CSF flow in and out of the cranium		TR=6.7 ms and TE=3.6 ms		1.72 × 1.72 × 10.0		110 × 110 mm		2 × 2 min, 8 s		Velocity encoding value=10 and 80 cm/s; FA=4°	

^a^FOV: field of view.

^b^MPRAGE: Magnetization-Prepared Rapid Acquisition Gradient Echo.

^c^Default imaging protocol at the scanner using standard Philips software.

^d^TR: repetition time.

^e^TE: echo time.

^f^FA: flip angle.

^g^FLAIR: fluid-attenuated inversion recovery.

^h^WMH: white matter hyperintensity.

^i^TI: inversion time.

^j^TSE: turbo spin echo.

^k^SWI: susceptibility-weighted imaging.

^l^DWI: diffusion-weighted imaging.

^m^BOLD-CSF: blood oxygen level–dependent signal and cerebrospinal fluid coupling.

^n^CSF: cerebrospinal fluid.

^o^MR: magnetic resonance.

^p^pCASL: pseudocontinuous arterial spin labeling.

^q^GRE: gradient-recalled echo readout.

^r^CSF-STREAM: CSF-selective T2-prepared readout with acceleration and mobility encoding.

**Figure 2:**
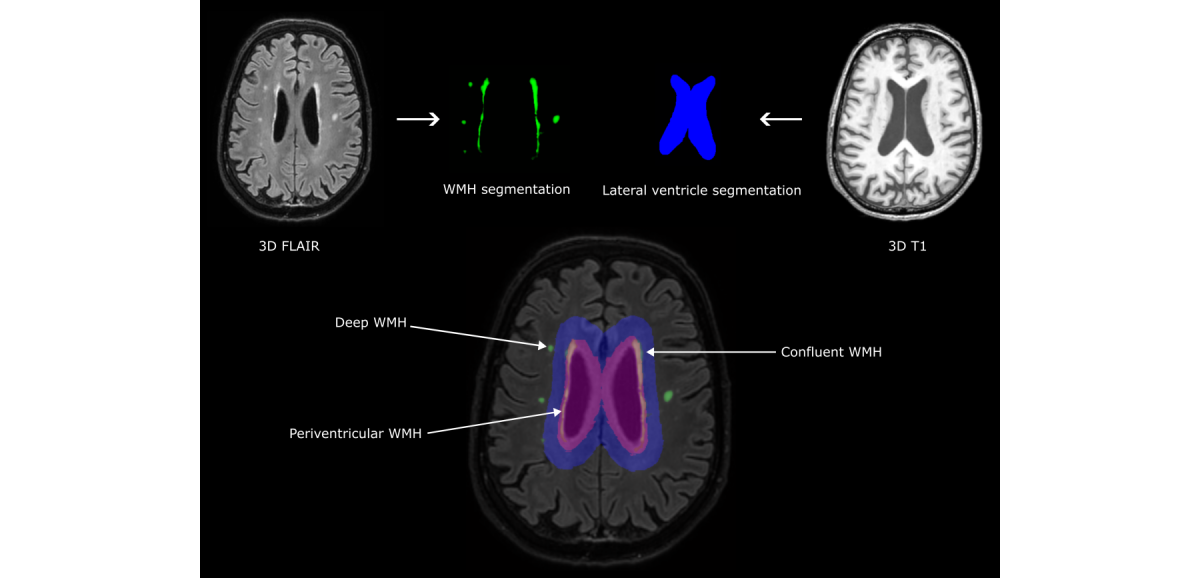
Overview of the magnetic resonance imaging (MRI) processing pipeline for white matter hyperintensity (WMH) shape. WMHs are automatically segmented from fluid-attenuated inversion recovery (FLAIR) MRI scans. T1-weighted scans are used to segment the lateral ventricles. The lateral ventricle masks are inflated and used to delineate between periventricular or confluent, and deep WMHs. The WMH shape markers are then calculated on individual lesions.

**Figure 3:**
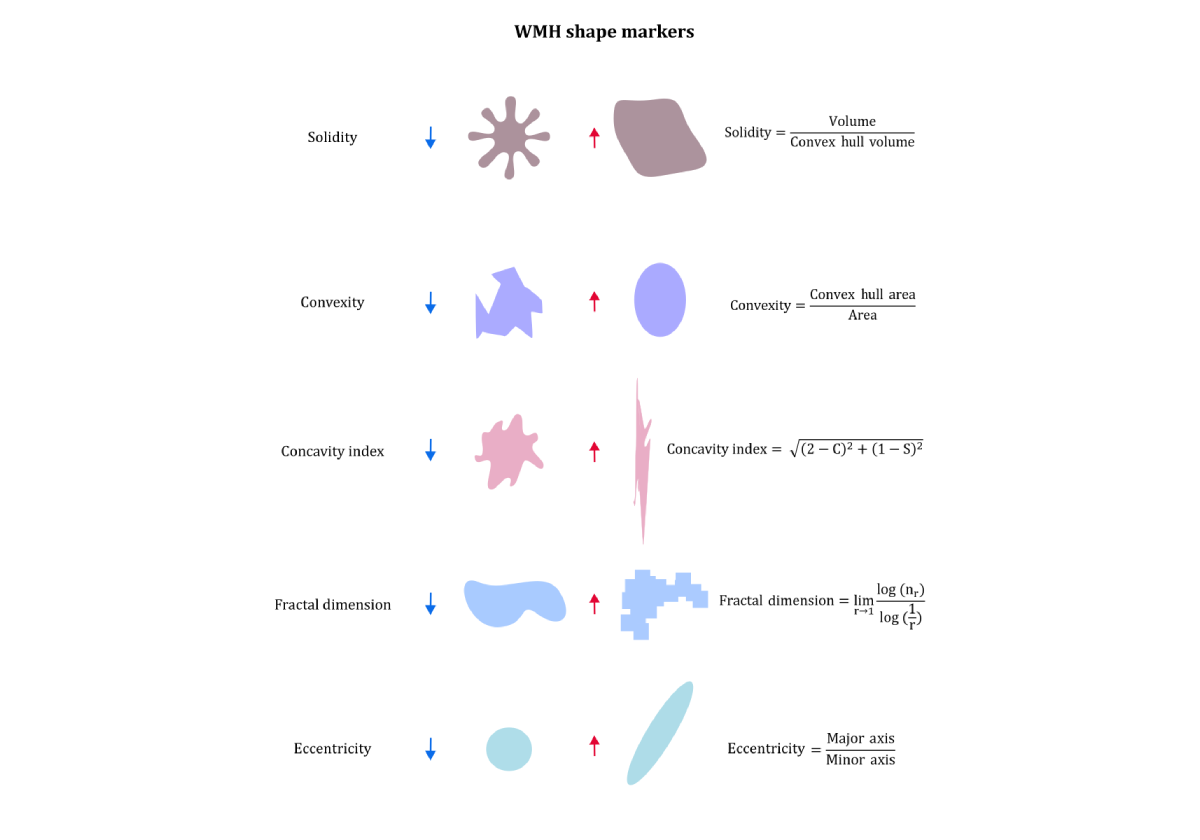
Examples of shapes with high or low values of different shape markers. Solidity, convexity, concavity index, and fractal dimension are calculated for periventricular and confluent white matter hyperintensities (WMHs). Eccentricity and fractal dimension are the shape markers that are calculated for deep WMHs.

Conventional (3T) brain MRI scans will be used to determine global and functional markers of cerebral SVD, such as WMH volume and presence of lacunes, microbleeds and superficial siderosis (on a 3D T1-weighted, 3D fluid-attenuated inversion recovery [FLAIR], susceptibility-weighted imaging, and diffusion-weighted imaging scan), and hemodynamics (flow territory mapping [41]). SVD markers will be assessed according to the second iteration of the Standard for Reporting Vascular Changes on Neuroimaging consensus criteria [42]. WMH shape will be calculated in the 3D space using an in-house developed pipeline, as described previously [18]. In brief, WMHs are segmented automatically from FLAIR MRI scans. Lateral ventricles are segmented from T1-weighted scans, and these ventricle masks are inflated by 3 mm and 10 mm. The inflated ventricle masks represent regional references for categorizing WMHs into periventricular, confluent, or deep subtypes. Examples of WMH segmentations and the shape markers are shown in Figures 2 and 3. Convexity, solidity, concavity index, and fractal dimensions will be determined based on the segmentations of the periventricular or confluent WMHs [18]. A lower convexity and solidity and higher concavity index and fractal dimension indicate more irregular shapes. For deep WMHs, fractal dimensions and eccentricity will be calculated [18]. Lower eccentricity indicates a rounder shape, and a higher fractal dimension indicates a more irregular shape of deep WMHs. The formulas of the WMH shape markers are shown in Figure 3. Moreover, total WMH volume, as well as the volumes of periventricular, confluent, and deep WMHs, will be calculated in milliliters.

White matter structural integrity will be measured using a magnetic resonance fingerprinting (MRF) sequence [43] and an inhomogeneous magnetization transfer (ihMT) scan [44]. A gradient-spoiled MRF [43] sequence with an optimized flip angle pattern for T1 and T2 quantification [45] was used. The MRF sequence is sensitive to both T1 and T2 relaxation effects in a time-efficient manner. This allows for quantitative T1 and T2 measurements and quantification of underlying magnetization fractions of the tissue present in the brain [46,47]. A 3D gradient echo–based ihMT sequence was acquired in which we used interleaved off-resonance radio-frequency saturation pulses to isolate dipolar effects, which have been linked to myelin [44]. These 2 sequences quantify underlying white matter tissue properties within and outside of the WMHs to better understand their pathological processes in vivo and see whether these are related to a more irregular WMH shape.

A high temporal resting-state functional MRI sequence will be obtained, with its first obtained slice strategically positioned below the fourth ventricle. This will allow for probing of the inflow dynamics of CSF into the brain and correlating these with gray matter BOLD signals [48]. Altered BOLD-CSF coupling might signal impaired drivers of brain clearance activity, such as a decreased driving force of CSF motion due to arteriolar vasomotion, especially when found in relation to lower cognitive scores or more severe SVD burden [49,50].

Phase-contrast sequences will be used, first, to quantify arterial blood flow in the feet-head direction of blood flowing into the brain (Q flow) and, second, as angio-surveys to facilitate the placement of the labeling plane perpendicular to brain-feeding arteries and measure the offset distance between arteries (ie, left-right and anterior-posterior) for flow territory mapping. Q flow can also be used to measure blood flow velocities. Flow territory mapping is achieved through arterial spin labeling MRI. It can demarcate perfusion territories by changing the labeling efficiency within the labeling plane in the right-left and anterior-posterior directions over sequential arterial spin labeling measurements.

Apart from BOLD-CSF coupling, these advanced 3T MRI sequences are secondary to the main aims and will provide additional information on the tissue and perfusion properties of WMHs.

**Figure 4:**
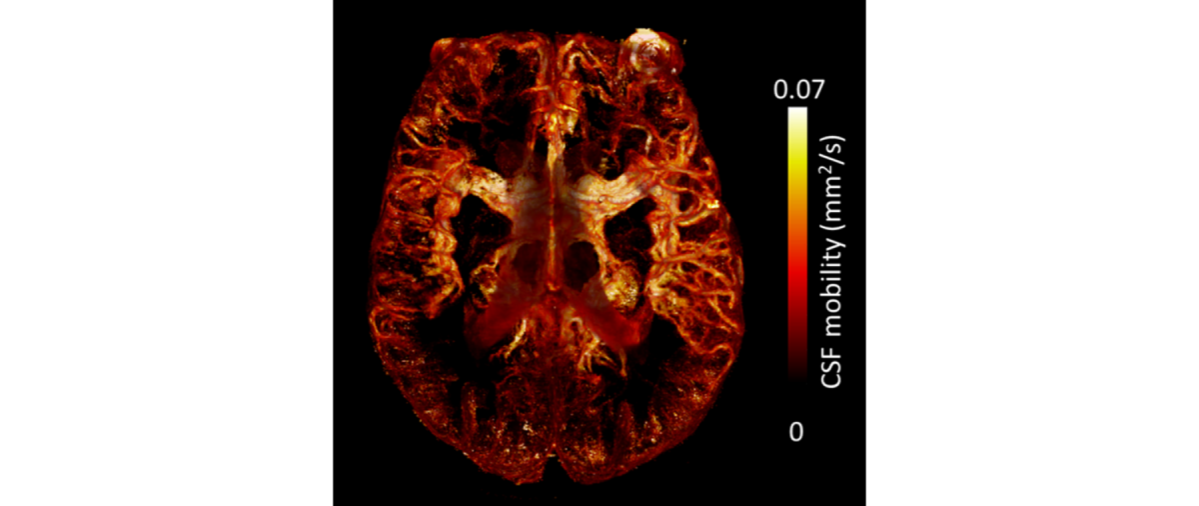
Example of an image obtained using the cerebrospinal fluid (CSF)–selective T2-prepared readout with acceleration and mobility encoding sequence as a measure of brain clearance activity. The image shows an example of a CSF mobility map from a White Matter Hyperintensity Shape and Brain Clearance Study (WHIMAS) patient.

Ultrahigh-field (7T) brain MRI scans will be used to determine WMH shape [18] (solidity, convexity, concavity index, fractal dimension, and eccentricity) and other markers of cerebral SVD in and surrounding the WMHs, such as enlarged PVS, (cortical) microinfarcts, and microbleeds (on a T1-weighted, T2-weighted, FLAIR, and T2*-weighted scan). We will investigate the effect of a higher resolution and increased contrast on the WMH shape calculations. Moreover, a recently implemented MRI technique with high resolution called CSF-selective T2-prepared readout with acceleration and mobility encoding (CSF-STREAM) provides detailed whole-brain imaging of CSF mobility, from subarachnoid spaces down to the level of PVS as a proxy for brain clearance activity [51]. An example of measurements obtained using this technique can be found in Figure 4. Moreover, we will measure CSF and blood flow at the level of the foramen magnum using phase-contrast MRI. Heart rate and respiratory signals will be measured during the scans (3T and 7T MRI) using standard vendor-supplied equipment, namely, a respiratory belt and pulse oximeter.

Patients are asked to lie as still as possible during scanning. If any discomfort is felt, they may move that body part slightly during breaks in between acquisitions, but we emphasize that they should not move their head, neck, or shoulders in doing so. Between the 3T and 7T scans, an hour break is planned. Furthermore, patients are asked beforehand if they have neck or back pain that prevents them from laying supine for extended periods. If that is the case, they are excluded as the risk of not completing the MRI scans is high.

CSF-STREAM consists of 7 subscans of 2 minutes, 45 seconds, limiting the effect of motion during each subscan. The 7 subscans will be coregistered with the first scan to correct for motion. If high motion occurs during one of the subscans, resulting in strong image artifacts, the corresponding scans will be excluded from the analysis. BOLD-CSF coupling is a very fast BOLD echo-planar imaging sequence and can be motion corrected retrospectively. The ihMT scan consists of 5 acquisitions with varying on- and off-resonance radio-frequency pulses. These 5 images will be motion corrected to each other similar to the CSF-STREAM strategy or excluded if they show high within-image motion. Furthermore, the large voxel sizes will somewhat mitigate motion blurring.

The T2* gradient-echo readout has a navigator that can prospectively mitigate motion during acquisition.

### Statistics

Multivariable regression analyses will be conducted to relate periventricular and deep WMH volume and shape (solidity, convexity, concavity index, fractal dimension, and eccentricity) to brain clearance markers (eg, peak BOLD-CSF coupling magnitude, CSF mobility and flow, and PVS count) and white matter integrity (magnetization transfer ratio and mean T1 and T2 of the white matter maps). Furthermore, the association between WMH shape and brain clearance markers and *z*-transformed cognitive domain scores (memory, executive function, visuoconstruction, and processing speed) will be studied via multivariable regression analyses.

Secondary analyses could include association analyses with other SVD markers (presence of microbleeds, lacunes, and small recent subcortical infarcts).

Potentially confounding demographic variables such as age, sex, level of education, intracranial volume, and vascular risk factors will be included as covariates for subsequent analyses when appropriate.

Statistical assumptions such as linearity and normality of residuals will be checked using *Q*-*Q* plots, histograms, and other distribution measures. If applicable, appropriate nonparametric tests will be used, or the variables will be log-transformed. For the main exploratory analyses, the significance will be set at *P*<.05. Correction for multiple testing will be performed when appropriate.

### Ethical Considerations

The WHIMAS study (NL78641.058.21) was approved by the Medical Ethics Committee Leiden The Hague Delft (P21.114) and is registered on ClinicalTrials.gov (NCT06010511). All participants will provide written informed consent before any experiments. Data will be anonymized and, after completion of data collection, transmitted to a secure repository with safeguards in place to protect confidentiality. Participants will receive travel compensation.

## Results

Patient inclusion started in January 2023, and study enrollment of patients is expected to finish in the second quarter of 2027. The main results are expected to be published in the first quarter of 2028.

## Discussion

In the WHIMAS study, we aim to study the link between WMHs, and especially their shape, and brain clearance and other MRI markers on ultrahigh- and high-field brain MRI and show whether WMH shape and brain clearance markers are associated with cognitive functioning in older adults with memory complaints. A more irregular WMH shape is defined as lower solidity, lower convexity, higher concavity index, and higher fractal dimension of periventricular WMHs. This more irregular WMH shape can be described as a reduced “smoothness” of the WMH outer border. Our hypothesis is that a more irregular WMH shape is related to brain clearance abnormalities (captured using MRI, eg, altered CSF flow in the fourth ventricle and PVS) and disease severity (captured through other SVD markers). Furthermore, we also hypothesize that a more irregular WMH shape and brain clearance abnormalities are related to reduced cognitive functioning (memory, executive function, visuoconstruction, or processing speed domain). We expect to find the most significant associations in periventricular WMH shape as, in a previous study, a more irregular periventricular WMH shape was associated with cerebral SVD progression and cognition over a 5-year follow-up [52,53], as well as with an increased long-term dementia risk [18].

SVD in an aging population has a heterogeneous underlying pathology that current MRI markers do not accurately capture. Novel 7T brain MRI markers provide a window of opportunity to study early structural changes and potential determinants of SVD. WMH shape is a relatively novel MRI marker, and previous studies have shown prognostic potential [18,19]. However, the exact microstructural changes within or surrounding WMHs or potential determinants related to WMH shape variations remain unknown. Furthermore, impaired brain clearance via the recently discovered brain clearance system may be another early change or potential cause of SVD [54].

The WHIMAS study will generate data that can be used to postulate underlying mechanisms related to WMH shape variations as we will study the association between a more irregular WMH shape and advanced structural and functional markers of cerebral SVD. We expect to gain a better understanding of the structural correlates of WMH shape variation using and combining the advanced measurements of 7T MRI. WMH shape has previously been related to an increased long-term dementia risk [18] and increased long-term stroke and mortality risk [19]. In our study, we expect to find that a more irregular WMH shape will be related to disease severity (captured via other SVD markers) and cognition (memory, executive function, visuoconstruction, and processing speed). Moreover, we expect to capture WMH shape and other structures that may influence shape (such as veins) better through 7T MRI due to an increased spatial resolution. This will allow for a more precise investigation of WMH shape in relation to other SVD markers and structural changes.

Animal studies suggest that dysfunction of the brain clearance system plays a major role in the initiation and progression of not only neurodegenerative pathologies but also SVD [54]. The novel noninvasive markers of brain clearance that we will use in our study will allow us to assess brain clearance in vivo in a noninvasive way. In our study, we expect to find an association between CSF mobility and disease severity. Moreover, the BOLD-CSF coupling has been found to be reduced in patients with Alzheimer disease [50] and cerebral amyloid angiopathy [51]. Therefore, we expect that the BOLD-CSF coupling will be reduced in our patient population and will show an association with disease severity.

Biomarkers early in the disease process of cerebral SVD are extremely important as they may represent a basis for future patient selection for lifestyle interventions and outcome markers of treatment trials aimed at the prevention or treatment of dementia. The results of our study will contribute to the body of evidence for novel brain MRI markers that could hopefully serve as early biomarkers for SVD.

The strengths of our study include the specific patient population and the use of advanced ultrahigh-field 7T state-of-the-art MRI techniques in combination with advanced image processing techniques largely developed within our research group. This study serves to steer future investigations and could be extended into a longitudinal study. This study has several limitations that we would like to address. Undergoing 2 MRI scans of 1 hour each in combination with a neuropsychological assessment can be overwhelming for the targeted patient group. This could limit the inclusion rates and bias our sample toward either healthier or more highly motivated participants. Furthermore, many of the advanced MRI techniques are highly motion sensitive, which may increase the total number of participants needed for the analysis.

In conclusion, in the WHIMAS study, we aim to find brain MRI changes that could represent early determinants of or changes in cerebral SVD and that should be assessed in more detail in future studies in healthy older adults. These markers early in the disease process of SVD could be extremely important as they may represent a basis for future patient selection for lifestyle interventions or for treatment trials aimed at the prevention of dementia.

### Data Availability

Only one scan of a single patient was used for this paper as an example of the techniques used. In-house developed magnetic resonance imaging sequences can be shared with interested research groups when proper collaboration agreements are in place.

## Abbreviations

BOLD: blood oxygen level–dependent
CSF: cerebrospinal fluid
CSF-STREAM: cerebrospinal fluid–selective T2-prepared readout with acceleration and mobility encoding
FLAIR: fluid-attenuated inversion recovery
ihMT: inhomogeneous magnetization transfer
LUMC: Leiden University Medical Center
MRF: magnetic resonance fingerprinting
MRI: magnetic resonance imaging
SVD: small vessel disease
WHIMAS: White Matter Hyperintensity Shape and Brain Clearance
WMH: white matter hyperintensity
